# Isoliquiritigenin attenuates septic acute kidney injury by regulating ferritinophagy-mediated ferroptosis

**DOI:** 10.1080/0886022X.2021.2003208

**Published:** 2021-11-18

**Authors:** Yun Tang, Haojun Luo, Qiong Xiao, Li Li, Xiang Zhong, Jiong Zhang, Fang Wang, Guisen Li, Li Wang, Yi Li

**Affiliations:** a Department of Nephrology, School of Medicine, Sichuan Academy of Medical Science and Sichuan Provincial People’s Hospital, Sichuan Clinical Research Center for Kidney Diseases, Clinical Immunology Translational Medicine Key Laboratory of Sichuan Province, University of Electronic Science and Technology of China, Chengdu, China; b Laboratory of Pathology, West China Hospital, Sichuan University, Chengdu, China

**Keywords:** Acute kidney injury, isoliquiritigenin, ferroptosis, ferritinophagy, lipid peroxidation

## Abstract

Defined differently from apoptosis, necrosis, and autophagy, ferroptosis has been implicated in acute kidney injury (AKI) such as ischemia-reperfusion injury induced AKI, folic acid caused AKI and cisplatin induced AKI. However, whether ferroptosis is involved in LPS induced AKI could be remaining unclear and there is still a lack of therapies associated with ferroptosis in LPS induced AKI without side effects. This study aimed to elucidate the role of isoliquiritigenin (ISL) in ferroptosis of LPS-induced AKI. We used LPS to induce renal tubular injury, followed by treatment with ISL both *in vitro* and *in vivo*. Human renal tubular HK2 cells were pretreated with 50 μM or 100 μM ISL for 5 h before stimulation with 2 μg/mL LPS. Mice were administered a single dose of either 50 mg/kg ISL orally or 5 mg/kg ferroptosis inhibitor ferrostatin-1 intraperitoneally before 10 mg/kg LPS injection. We found that LPS could induce mitochondria injury of renal tubular presented as the shape of mitochondria appeared smaller than normal with increased membrane density and are faction or destruction of mitochondrial crista through scanning electron microscope. Ferrostatin-1 significantly protected mice against renal dysfunction and renal tubular damage in LPS-induced AKI. ISL inhibited Fe^2+^ and lipid peroxidation accumulation in LPS-stimulated HK2 cells. It also increased the expression of GPX4 and xCT, reduced the expression of HMGB1 and NCOA4 then attenuated mitochondria injury in renal tubular following LPS stimulation. These results indicated the potential role of ISL against ferritinophagy-mediated ferroptosis in renal tubular following LPS stimulation.

## Introduction

Acute kidney injury (AKI) is a major consequence of sepsis that affects renal tubular epithelial cells. It is typically associated with a high in-hospital mortality rate (50–60%) that increases among patients with sepsis (70–80%) [1–3]. Patients with AKI require dialysis and a transfer to the intensive care unit (ICU), presenting challenging circumstances from sanitary and economic points of view [4–6]. AKI favors progression to chronic kidney disease (CKD), increases medium- and long-term cardiovascular morbimortality, as well as causes permanent dialysis dependence [4]. Treatment and prevention strategies for patients with AKI continually pose a key challenge to the intensive care physician.

The pathophysiology of AKI in sepsis is complex, but it always causes part from systemic inflammation, nephrotoxins, and hemodynamic alterations [7]. AKI is also an acute cell death disease of renal tubular cells that occurs through multiple regulated cell death mechanisms, including necrosis, apoptosis, autophagy, and so on [8,9]. However, there is still a lack of effective therapies without side effect for cell death of renal tubular in septic AKI. Isoliquiritigenin (ISL) is a bioactive component isolated from the roots of the Chinese traditional herb licorice [10]. ISL performs useful biological functions owing to its anti-inflammatory, anti-oxidative, anti-nephritic, and anti-cancer properties [11]. LPS induced AKI mice model was commonly used to mimic septic AKI. To mimic the septic AKI, we used a LPS induced AKI mice model. This study was planned to elucidate the potential role of ISL in cell death of renal tubular in LPS-induced AKI. The present study aimed at investigating the potential role of ferroptosis in LPS-induced AKI and understanding the protective mechanism of ISL.

## Materials and methods

### Chemicals and reagents

Isoliquiritigenin was purchased from MedChemExpress (HY-N0102, Princeton, NJ). *Escherichia coli* O111:B4 LPS was obtained from Sigma-Aldrich (L4130, Sigma, St. Louis, MO).

### Animal model

All procedures were conducted in accordance with the guidelines for animal care and use from the National Institutes of Health. Male C57BL/6 mice (aged 6–8 weeks and weighing 22–25 g) were obtained from the Experimental Animal Center, Sichuan Provincial People’s Hospital, and were fed a standard laboratory diet. LPS and ISL were dissolved in normal saline and 0.5% Tween-20/saline, respectively. AKI mice were developed by intraperitoneal (*i.p.*) LPS injection. A total of 30 mice were randomly divided into six groups (*n* = 5): control, ISL, Fer, LPS, LPS plus ISL, and LPS plus Fer. An intraperitoneal injection of LPS (10 mg/kg) was made to induce septic AKI. ISL was administered *via* gavage at 50 mg/kg 30 min before LPS injection. Mice were dosed intraperitoneally with Fer (Ferrostatin-1, SML0583, Sigma-Aldrich, St. Louis, MO) at 5 mg/kg. Mice were sacrificed by cervical dislocation 8 h after LPS injection. Kidney tissue and serum samples were collected concurrently.

### Cell culture and treatments

Human kidney epithelial tubular cell line HK2 (ATCC® CRL-2190™) was grown in DME/F-12 medium (SH30023.01, HyClone, Logan, UT), supplemented with 10% FBS (Gibco, Life Technologies, Lofer, Austria), 100 units/mL penicillin, and 100 units/mL streptomycin (1705694, HyClone, Logan, UT), at 37 °C in a 5% carbon dioxide humidified incubator. Cells were treated with 50 μM or 100 μM ISL for 5 h, before septic AKI was induced using 2 μg/mL LPS. Cells were collected 24 h after LPS inducing. Cell experiments were repeated three times.

### Detection of Fe^2+^ and lipid peroxides using fluorescent probes

HK2 cells were seeded in a cell culture dish (801002, Nest Scientific, Woodbridge Township, NJ) containing DME/F-12 medium and cultured overnight at 37 °C in a 5% CO_2_ incubator. Cells were then treated with 1 μM FerroOrange (F374, Dojindo, Japan), 1 μM Liperfluo (L248, Dojindo, Japan), or 5 μM mito-Ferrogreen (M489, Dojindo, Japan) for 30 min at 37 °C, and visualized with blindness using a confocal microscope (LSM 800, Zeiss, San Diego, CA) equipped with a 60× oil immersion objective.

### Immunohistochemical analyses

Formalin-fixed renal tissues were embedded in paraffin and cut into sections 2-μm thick. The sections were routinely deparaffinized and rehydrated, and tissues were subjected to immunohistochemical staining for GPX4 (1:200; ab125066, Abcam, Cambridge, MA), xCT (5 µg/mL; NB300-318, Novus Biologicals, Littleton, CO), and NCOA4 (1:100, abs134557, Absin Bioscience, Shanghai, China), in strict accordance with the manufacturers’ instructions. Renal tissues stained for GPX4, NCOA4, and xCT were assessed by a renal pathologist in a blinded fashion. Images were captured with a light microscope under an original magnification of ×400.

### Electron microscopy

Tissue samples were prepared for transmission electron microscopy (TEM) by blocking the kidney cortex in Fixative (P1126, Servicebio, Beijing, China) at 4 °C for at least 2–4 h and washed three times in 0.1 M PBS (15 min per wash). Kidney tissues were post-fixed with 1% OsO4 in 0.1 M PBS (pH 7.4) for 2 h at 18–22 °C, dehydrated in graded concentrations of ethanol, and embedded in paraffin by baking in an oven at 60 °C for 48 h. Ultrathin sections (60–80 nm in thickness) were stained with uranyl acetate and lead citrate and examined *via* TEM (HT7700, HITACHI, Tokyo, Japan).

### Hematoxylin and eosin (H&E) and periodic acid-Schiff (PAS) staining

Formalin-fixed renal tissues were embedded in paraffin and cut into sections 2-μm thick. The sections were stained with H&E and PAS solutions. Images were captured using light microscope under an original magnification of ×400. The histologic diagnosis of renal tubular injury based on previous study, including epithelial simplification, loss of brush border, apical blebbing, epithelial cell sloughing, tubular dilatation, and cast formation. Renal tissue sections were assessed by two renal pathologists in a blinded fashion. The damage score was based on the results of PAS staining according to previous study: ‘0’ for all normal tubules; ‘1’ for <10% injury tubules; ‘2’ for 10–25% injury tubules; ‘3’ for 26–75% injury tubules; and ‘4’ for >75% injury tubules [12].

### Assessment of renal function

Blood samples were centrifuged at 3000 rpm for 10 min at 4 °C. The serum was collected and stored at −80 °C. Blood urea nitrogen (BUN) and serum creatinine (SCr) were detected using the BUN (C013-2) and Creatinine (C011-2) Assay Kits (Jiancheng, Nanjing, China), respectively.

### Western blotting

Proteins in kidney tissues and HK2 cells were extracted using RIPA lysis buffer (#P0013B, Beyotime, Shanghai, China) containing 1% PMSF (P0100, Solarbio, Beijing, China). The proteins were separated by 10% SDS-PAGE and transferred to PVDF membranes (R7CA6580A, Thermo Fisher Scientific, Waltham, MA). The membranes were probed overnight with GPX4 (1:1000, ab125066, Abcam, Cambridge, MA), and NCOA4 (1:1000, abs-134557, Absin, Shanghai, China). The membranes were respectively incubated with HRP-labeled goat anti-rabbit IgG (1:5000, 511203, Zen BioScience, Research Triangle Park, NC) and HRP labeled goat anti-mouse IgG (1:5000, 511103, Zen BioScience). Bands were detected using Immobilon Western Chemilum HRP Substrate (Catalogue number WBKLS0500, EMD Millipore, Burlington, MA), and protein levels were normalized against β-actin (1:5000; HRP-60008, Proteintech, Rosemont, IL).

### Measurement of nitric oxide

Serum samples and cell supernatant were centrifuged by 3000 rpm for 10 min to measure the secretion of nitric oxide. The levels of nitric oxide were detected by total Nitric Oxide Assay Kit (S0021S, Beyotime, Shanghai, China) involving Griess Reagent. According to instruction from manufacturer, 50 µl murine serum samples and cell supernatant samples were respectively added into 96-well plates. Then the samples reacted with adequate Griess Reagent solutions. Then absorbance of 540 nm was recorded by a microplate reader (#680, BioRad, Hercules, CA).

### Detection of malondialdehyde (MDA)

For MDA detection, renal tissues and HK2 cells were lytic by RIPA lysis buffer and centrifuged by 3000 rpm for 10 min. The protein concentration was detected by Bradford Protein Assay Kit (Beyotime, Beijing, China) used for the calculation of MDA. The level of MDA was measured by MDA assay kit (A003-1-2, Jiancheng, Nanjing, China) involving the TBA method. According to the instruction of MDA assay kit, the absorbance of 540 nm was recorded by microplate reader (#680, BioRad, Hercules, CA).

### Statistical analyses

Results were expressed as means ± standard deviation (SD). Statistical analyses were conducted by performing one-way ANOVA with post-hoc tests, using the Graph Pad Prism 8 software (GraphPad Software, La Jolla, CA). The Newman–Keuls multiple comparison test was used to compare differences. Differences between groups were deemed statistically significant at *p* < 0.05.

## Results

### ISL attenuated renal dysfunction and renal tubular damage in LPS-induced AKI

**Figure 1.**
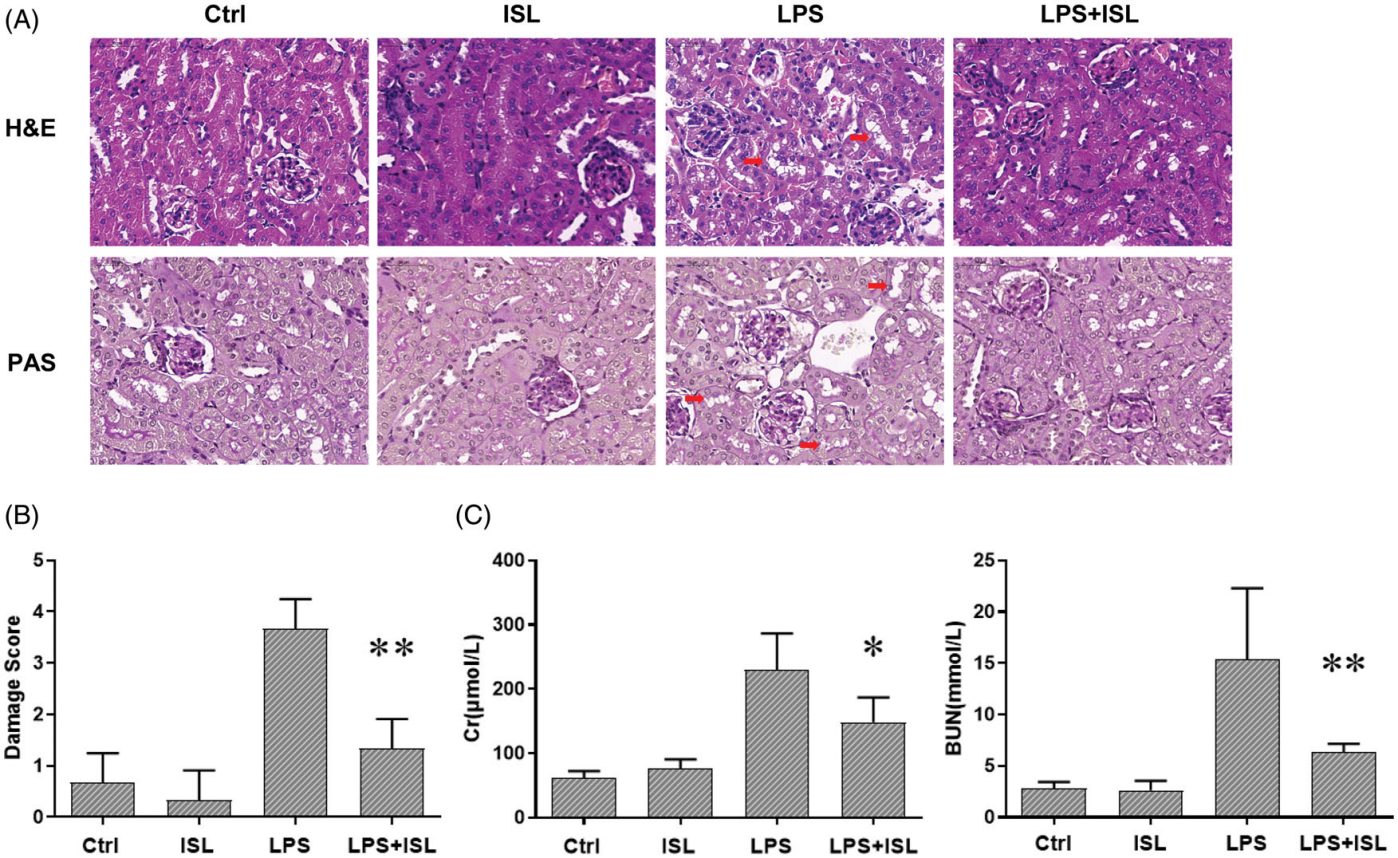
ISL attenuated pathological injury of murine kidney and renal dysfunction in the LPS-induced mouse model. LPS induce AKI mice models were developed by intraperitoneal (*i.p.*) LPS injection. A total of 30 mice were randomly divided into six groups (*n* = 5): control, ISL, Fer, LPS, LPS plus ISL, and LPS plus Fer. An intraperitoneal injection of LPS (10 mg/kg) was made to induce septic AKI. ISL was administered *via* gavage at 50 mg/kg 30 min before LPS injection. (A) H&E and PAS staining. (B) Damage score of renal tubular injury. (C) Murine renal function detection about SCr and BUN. ‘*’means compared with the LPS group and *p* < 0.05. ‘**’means compared with the LPS group and *p* < 0.01.

To validate the protective role of ISL in LPS-induced AKI, we treated mice with ISL followed by LPS injection. The results of H&E and PAS staining revealed ISL could attenuate pathological injury of mice kidney tissue after LPS injection (Figure 1(A)). To evaluate the histological changes in renal tubular, we analyzed damage score upon histological staining of murine kidney. The results of damage score showed that ISL could significantly decreased damage score after LPS injection. Following LPS injection, renal tubules developed a moderate to severe degree of injury, compared with the control group. ISL produced a significant protective role in kidney tissues, thereby potentially inhibited the murine kidney to develop a serious renal tubule injury (Figure 1(B)). Otherwise, LPS induced renal dysfunction in mice with a significant increase of SCr and BUN levels. However, ISL treatment reduced the levels of SCr and BUN in mice following LPS injection (Figure 1(C)).

### ISL reduced accumulation of MDA and nitric oxide after LPS induction

**Figure 2.**
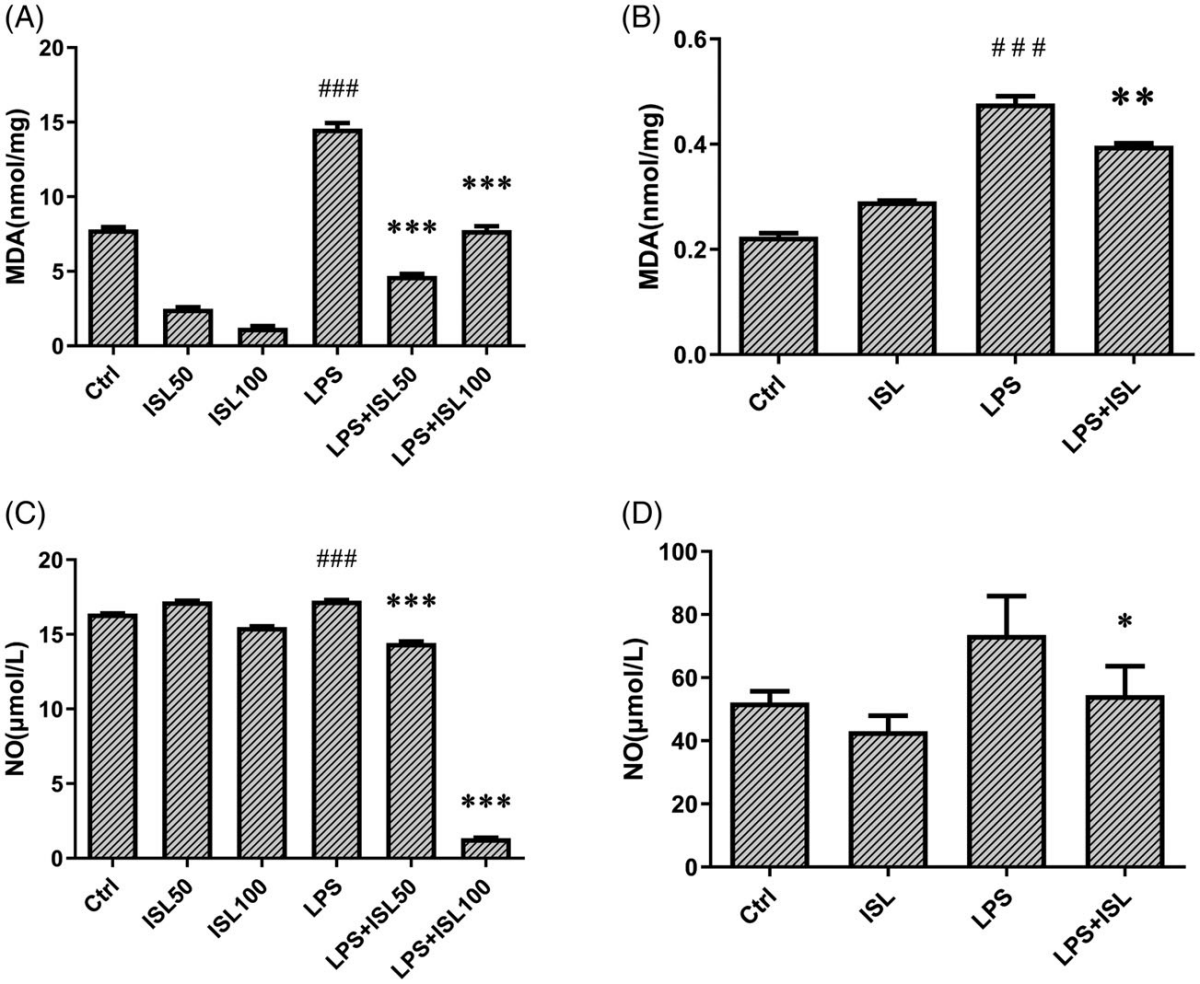
Measurement of MDA and nitric oxide after ISL treatment upon LPS induction. LPS induce AKI mice models were developed by intraperitoneal (*i.p.*) LPS injection. A total of 30 mice were randomly divided into six groups (*n* = 5): control, ISL, Fer, LPS, LPS plus ISL, and LPS plus Fer. An intraperitoneal injection of LPS (10 mg/kg) was made to induce septic AKI. ISL was administered *via* gavage at 50 mg/kg 30 min before LPS injection. HK2 cells were treated with 50 μM or 100 μM ISL for 5 h, before septic AKI was induced using 2 μg/mL LPS. Cells were collected 24 h after LPS inducing. And the cell experiments were repeated at three times. (A) MDA measurement of mice kidney tissue homogenate. (B) MDA measurement of HK2 cell homogenate. (C) Nitric Oxide Assay of murine serum. (D) Nitric Oxide Assay of HK2 cell supernatant. ‘*’ means compared with the LPS group and *p* < 0.05. ‘**’ means compared with the LPS group and *p* < 0.01. ‘***’ means compared with the LPS group and *p* < 0.001. ‘#’ means compared with the control group and *p* < 0.05. ‘##’ means compared with the control group and *p* < 0.01. ‘###’ means compared with the control group and *p* < 0.001.

Oxygen-free radicals could attack polyunsaturated fatty acids then trigger intracellular lipid peroxidation to induce cell death and injury. The amount of MDA could reflect the degree of intracellular lipid peroxidation and indirectly reflect the degree of cell injury. We observed that LPS could significantly increase the level of MDA in murine total kidney homogenates and cell supernatant of HK2 cells, compared to that of normal controls. However, ISL treatment could decrease the level of MDA in murine kidney homogenates and cell supernatant of HK2 cells after LPS stimulation (Figure 2(A–D)). The results of Griess Reagent assay showed that LPS could significantly increase the secretion of nitric oxide both in murine serum and cell supernatant of HK2 cells, compared to that of normal controls. However, ISL treatment could reduce the level of nitric oxide both in murine serum and cell supernatant of HK2 cells after LPS stimulation (Figure 2(E, F)).

### ISL inhibited Fe^2+^ and lipid peroxidation accumulation in LPS-stimulated cells

**Figure 3.**
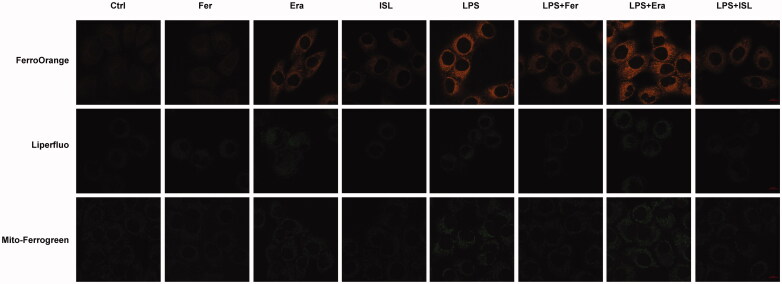
ISL inhibited Fe^2+^ and lipid peroxidation accumulation in LPS-stimulated cells. HK2 cells were treated with 50 μM or 100 μM ISL for 5 h, before septic AKI was induced using 2 μg/mL LPS. Cells were collected 24 h after LPS induction. FerroOrange was used as fluorescent probe to measure the level of Fe^2+^ in HK2 cells. Liperfluo was employed to detect the level of lipid peroxidation in HK2 cells. Mito-Ferrogreen could further detect Fe^2+^ ions in the mitochondria of HK2 cells. Magnification: ×60 oil.

We respectively treated HK2 cells with the ferroptosis inhibitor Ferrostatin-1 and agonist Erastin. FerroOrange was used as a fluorescent probe to measure the level of Fe**^2+^** in HK2 cells. Liperfluo was employed to detect the level of lipid peroxidation in HK2 cells. Mito-Ferrogreen could further detect Fe**^2+^** ions in the mitochondria of HK2 cells. Then we found that both LPS and Erastin could increase levels of Fe**^2+^** and lipid peroxidation in mitochondria of HK2 cells, whereas the levels of Fe**^2+^** and lipid peroxidation were decreased after treatment with Ferrostatin-1 or ISL (Figure 3).

### ISL increased the expression of GPX4 and xCT then attenuated mitochondria injury in renal tubular following LPS injection in mice

**Figure 4.**
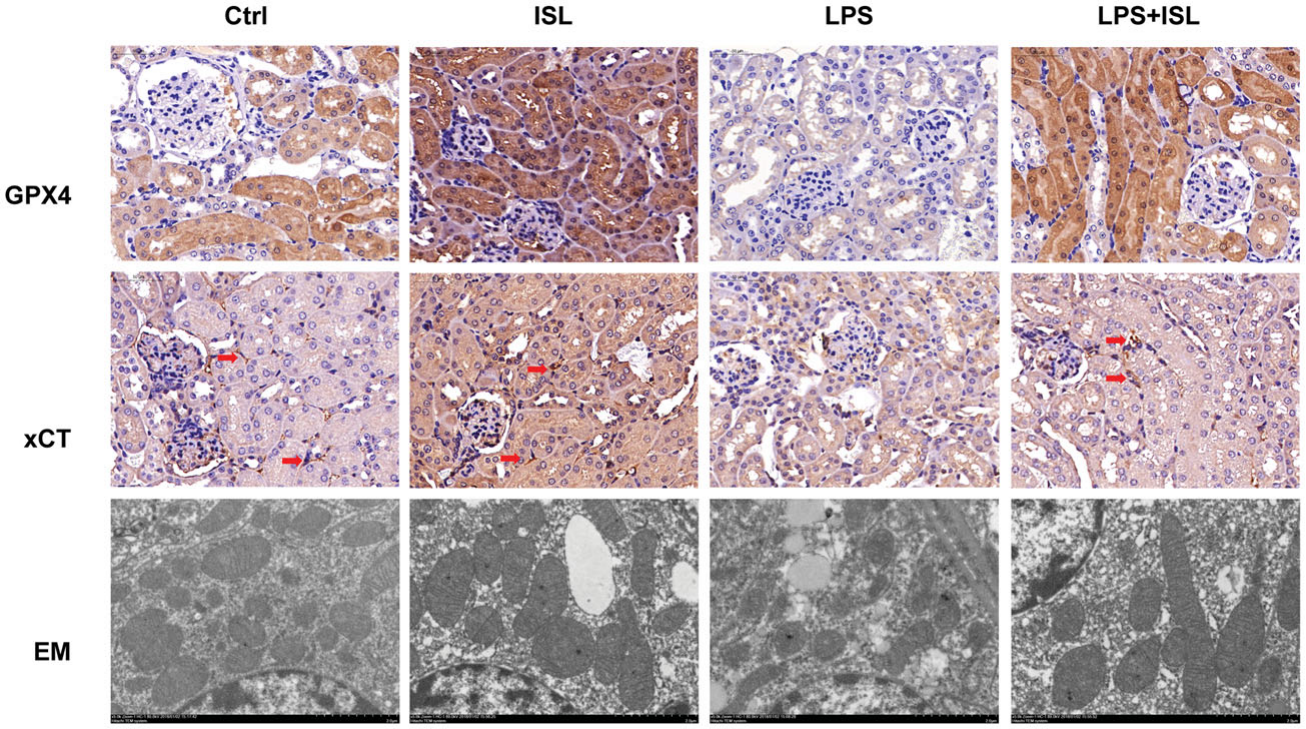
ISL increased the expression of GPX4 and xCT then attenuated mitochondria injury in renal tubular following LPS injection in mice. A total of 30 mice were randomly divided into six groups (*n* = 5): control, ISL, Fer, LPS, LPS plus ISL, and LPS plus Fer. An intraperitoneal injection of LPS (10 mg/kg) was made to induce septic AKI. ISL was administered *via* gavage at 50 mg/kg 30 min before LPS injection. Immunohistochemical staining in mice to detect GPX4 and xCT. And mitochondria analysis with scanning electron microscope. Magnification for immunohistochemical staining: ×400.

According to results of immunohistochemical staining of GPX4 and xCT, LPS injection could significantly inhibit the expression of GPX4 and xCT in murine renal tubular. However, ISL could increase the expression of GPX4 and xCT in murine renal tubular following LPS injection. Moreover, mitochondria analysis with scanning electron microscope showed that LPS could induce mitochondria injury of renal tubular presented as the shape of mitochondria appeared smaller than normal with increased membrane density and are faction or destruction of mitochondrial crista. Nevertheless, ISL could attenuate mitochondria injury in renal tubular following LPS injection in mice (Figure 4).

### Ferrostatin-1 protected mice against renal dysfunction and renal tubular damage in LPS-induced AKI

**Figure 5.**
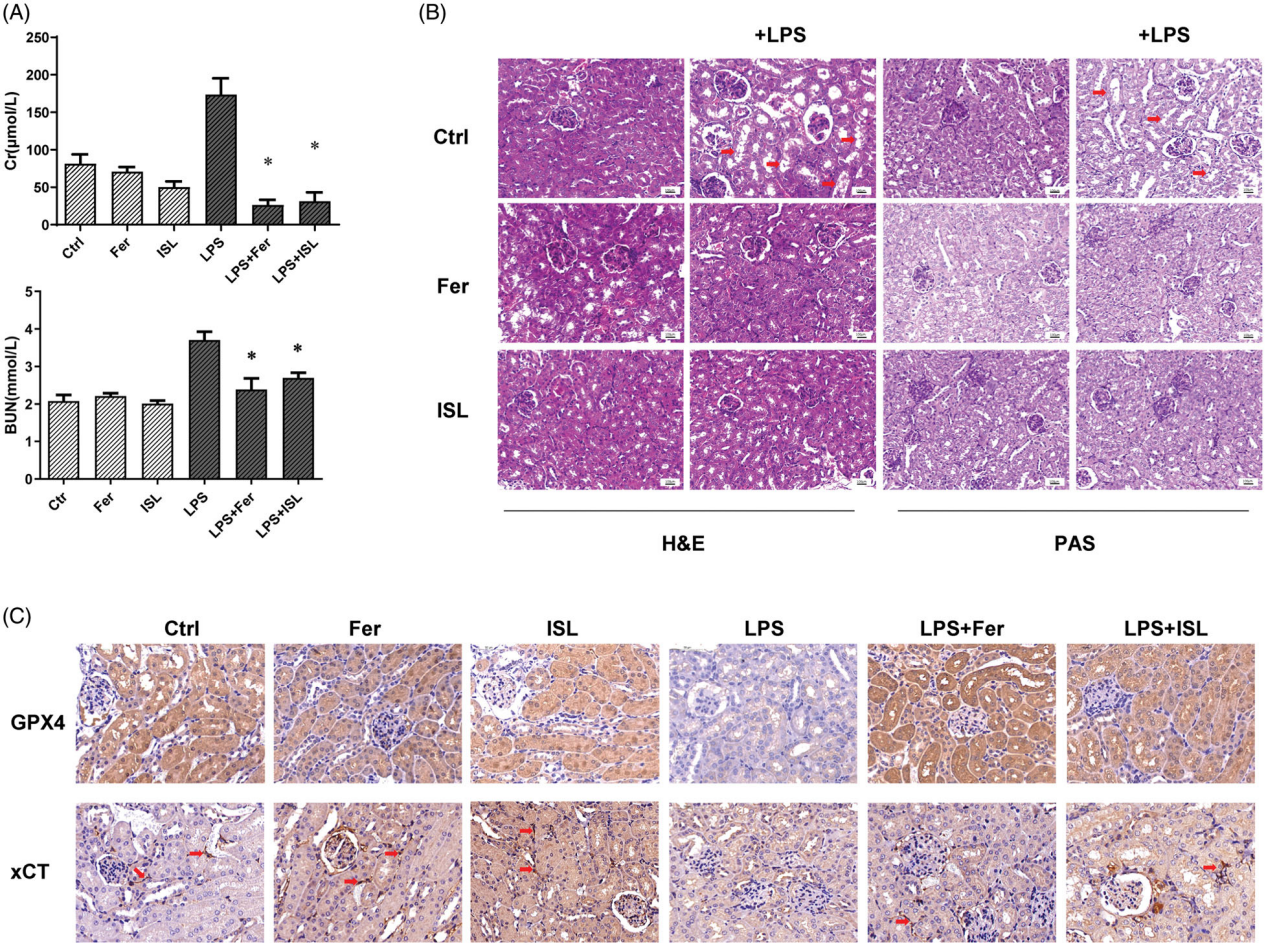
Ferrostatin-1 protected mice against renal dysfunction and renal tubular damage in LPS-induced AKI. Mice were administered a single dose of either 50 mg/kg ISL orally or 5 mg/kg ferroptosis inhibitor ferrostatin-1 intraperitoneally before 10 mg/kg LPS injection. (A) The level of SCr and BUN in mice. (B) The results of H&E and PAS staining. (C) Immunohistochemical staining with GPX4 and xCT in mice kidney. Magnification: ×400. ‘*’ means compared with the LPS group and *p* < 0.05.

LPS significantly increased the level of SCr and BUN in mice, compared to that of normal mice. However, both ISL and Ferrostatin-1 treatment could decrease the levels of SCr and BUN in mice following LPS injection (Figure 5(A)). The results of H&E and PAS staining also identified both ISL and Ferrostatin-1 treatment that could protect mice kidney against pathological injury of renal tubular following LPS injection (Figure 5(B)). The results of immunohistochemical staining with GPX4 and xCT showed both ISL and Ferrostatin-1 treatment that could obviously increase the expression of GPX4 and xCT in murine renal tubular following LPS injection (Figure 5(C)).

### ISL inhibited the expression of HMGB1 and increased the expression of GPX4 both *in vivo* and *in vitro* following LPS stimulation

**Figure 6.**
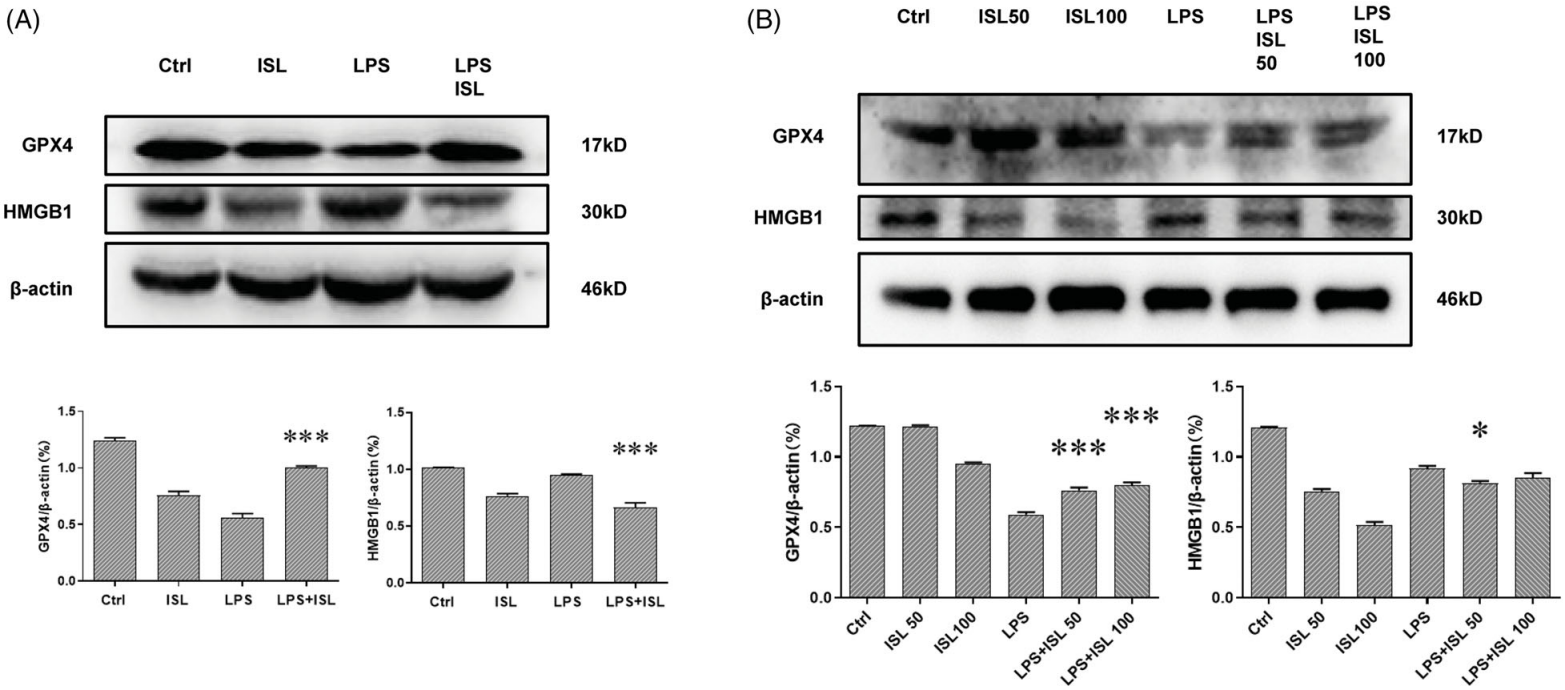
ISL inhibited the expression of HMGB1 and increased the expression of GPX4 both *in vivo* and *in vitro* following LPS stimulation. (A) Western blot assay about HMGB1 and GPX4 in murine kidney tissues. (B) Western blot assay about HMGB1 and GPX4 in HK2 cells.

We performed western blot assay upon murine kidney tissues and found that LPS injection could increase the expression of HMGB1 and reduce the expression of GPX4 in mice kidney, whereas ISL could inhibit the expression of HMGB1 and increase the expression of GPX4 in mice kidney following LPS injection (Figure 6(A)). LPS could also significantly increase the expression of HMGB1 and decrease the expression of GPX4 in HK2 cells. To further elucidate the effect of ISL on renal tubular, we observed that both 50 µM and 100 µM ISL could reduce the expression of HMGB1 and increase the expression of GPX4 in HK2 cells upon LPS stimulation (Figure 6(B)).

### ISL reduced the expression of NOCA4 both *in vivo and in vitro* upon LPS induction

**Figure 7.**
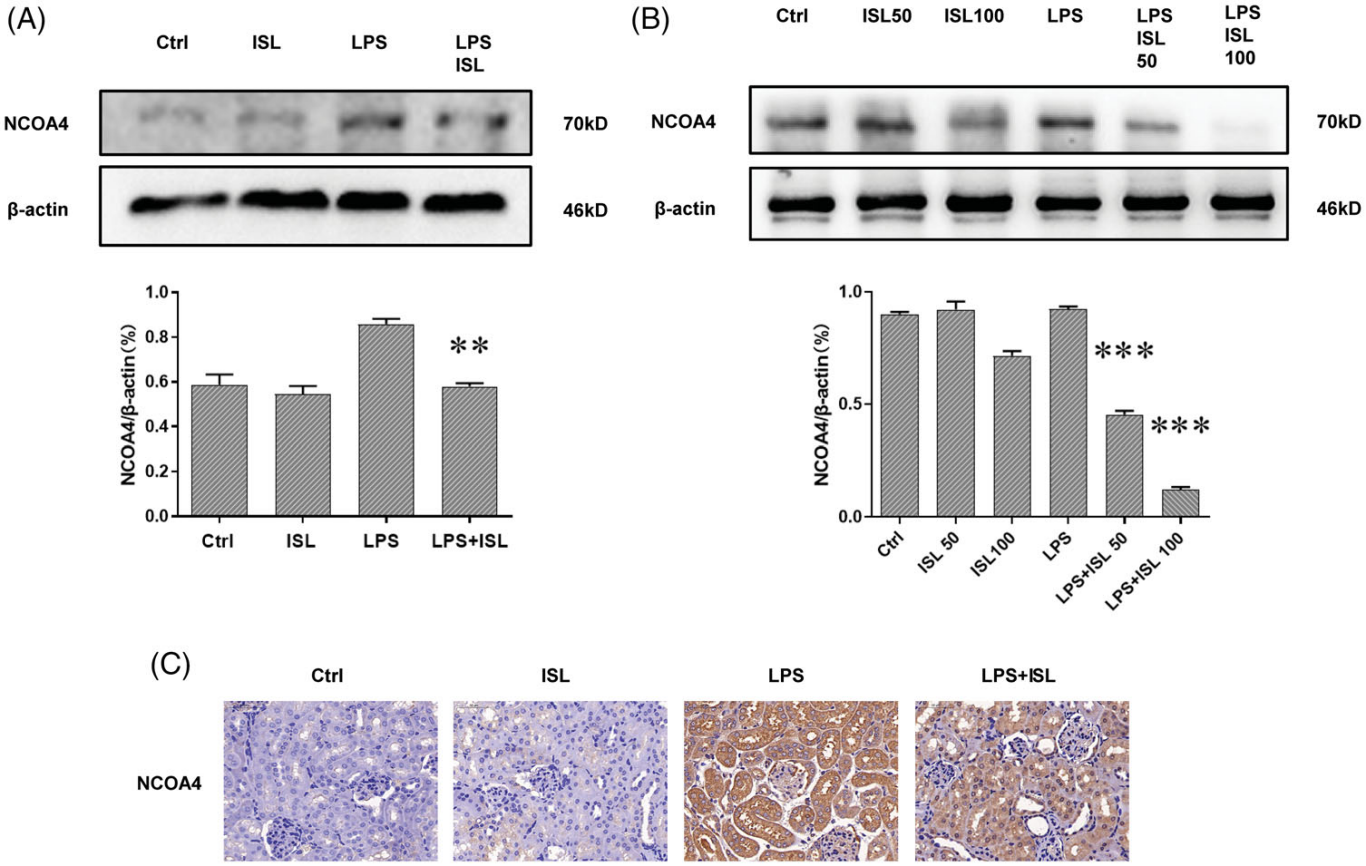
ISL reduced the expression of NOCA4 both *in vivo* and *in vitro* upon LPS induction (A) Western blot assay about NCOA4 in murine kidney tissues. (B) Western blot assay about NCOA4 in HK2 cells. (C) Immunohistochemical staining with NCOA4 in mice kidney tissues. Magnification for immunohistochemical staining: ×400.

The results of western blot with murine kidney tissues suggested that LPS injection could increase the expression of NOCA4 in mice kidney, whereas ISL could inhibit the expression of NCOA4 in mice kidney following LPS injection (Figure 7(A)). Although LPS could significantly increase the level of NOCA4 in HK2 cells, both 50 µM and 100 µM ISL could reduce the expression of NCOA4 in HK2 cells following LPS induction (Figure 7(B)). Immunohistochemical staining with NCOA4 showed LPS significant increase in the expression of NCOA4 in murine renal tubular. However, ISL treatment could decrease the expression of NCOA4 in murine renal tubular upon LPS injection (Figure 7(C)).

## Discussion

During LPS induced septic AKI, LPS could cause direct injury to renal tubular and induce cell death in renal tubules [13]. Regulated cell death of renal tubular in AKI always caused to sublethal and lethal damage of renal epithelial cells [8,14,15]. Originally defined in 2012 as an iron-dependent form of cancer cell death, ferroptosis could be different from apoptosis, necrosis, and autophagy [16]. It has been implicated in AKI such as ischemia–reperfusion injury induce AKI, folic acid caused AKI and cisplatin induced AKI [17–19]. However, whether ferroptosis is involved in LPS induced AKI could be remaining unclear yet. Our current study found that LPS could induce mitochondria injury of renal tubular presented as the shape of mitochondria appeared smaller than normal with increased membrane density and are faction or destruction of mitochondrial crista through scanning electron microscope. Ferroptosis inhibitor Ferrostatin-1 significantly protected mice against renal dysfunction and renal tubular damage in LPS-induced AKI. These results indicate the LPS might induce ferroptosis in renal tubular and inhibition of ferroptosis could protect mice from LPS induced AKI. Loss of activity of the lipid repair enzyme GPX4 and subsequent accumulation of lipid-based ROS, particularly lipid hydroperoxides always drove Ferroptosis [20]. And we observed that LPS could induce Fe^2+^ and lipid peroxidation accumulation in HK2 cells. It reduced the expression of GPX4 and xCT both in murine renal tissue and HK2 renal epithelial cells, suggesting ferroptosis of renal tubular in LPS induced AKI.

AKI is a multifactorial renal disease characterized by a rapid decline in renal function, that results in toxin accumulation and failure of other organs [8]. Clinically, sepsis is one of the main causes to AKI [8]. Effective intervention strategies need to be developed and implemented at the early stages of the disease. In a previous study, we established an LPS-induced AKI model to mimic septic AKI. And we found that ISL could attenuate extensive damage in renal tubules after LPS injection [21]. However, the role and mechanism of ISL in renal tubules involving ferroptosis in LPS induced AKI still remains to be further clarified. We then observed that ISL inhibited Fe^2+^ and lipid peroxidation accumulation in LPS-stimulated HK2 cells. It also increased the expression of GPX4 and xCT then attenuated mitochondria injury in renal tubular following LPS injection in mice. These indicated the potential role of ISL against ferroptosis in renal tubular following LPS stimulation.

**Figure 8.**
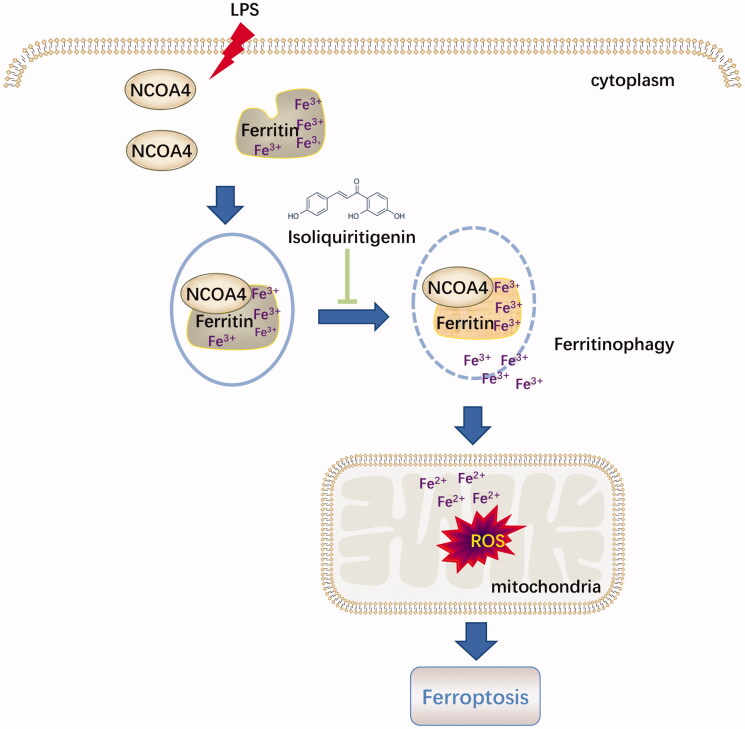
Diagram illustrated that the potential role of ISL against ferritinophagy-mediated ferroptosis in renal tubular following LPS stimulation.

Served as an endogenous endotoxin-like regulator in the process of LPS induced AKI, HMGB1 is a classic damage-associated molecular pattern molecule (DAMP) released by ferroptotic cells plays a location dependent role in promoting autophagy [22,23]. HMGB1-mediated ferritinophagy might enhance iron accumulation during ferroptotic damage [24]. Nuclear receptor coactivator 4 (NCOA4)-mediated ferritinophagy could maintain intracellular iron homeostasis by facilitating ferritin iron storage or release [25]. The current study showed that, accompanied with reduction of cytosolic Fe^2+^ and lipid peroxidation accumulation, ISL could significantly inhibit the expression of HMGB1 and increased the expression of NCOA4 both *in vivo* and *in vitro* following LPS stimulation. It suggested that ISL might attenuate LPS induced AKI by regulating ferritinophagy-mediated ferroptosis (Figure 8).

However, there could be some limitations for this study. Erastin could induced NCOA4-mediated ferritinophagy to promote ferroptosis, whereas not by RSL3 in HeLa cells [26]. The current study just observed the phenotype and effect of ISL about ferritinophagy-mediated ferroptosis in LPS induced AKI. In the next step of study, we will elucidate the precise mechanism of ISL about ferritinophagy-mediated ferroptosis in LPS induced AKI by treatment with both Erastin and ISL. For septic AKI, we preliminarily used LPS induced AKI model. We will further verify the precise mechanism of ISL about ferritinophagy-mediated ferroptosis a mouse model of lethal cecal ligation and puncture (CLP)-induced sepsis.

In the present study, we demonstrated the anti-inflammatory effects of ISL and Fer in LPS-induced AKI both *in vitro* and *in vivo*. It could be concluded that ISL might attenuate LPS induced AKI by regulating ferritinophagy-mediated ferroptosis.

## Abbreviations

AKI: acute kidney injury
CKD: chronic kidney disease
EM: electron microscope
Fer: Ferrostatin-1
GPX4: glutathione peroxidase 4
ISL: isoliquiritigenin
LPS: lipopolysaccharide
MDA: malondialdehyde
NCOA4: nuclear receptor coactivator 4
NO: nitric oxide
RN: regulated necrosis
ROS: reactive oxygen species
xCT: light-chain subunit of the cystine/glutamate transporter
